# The transcriptomic fingerprint of cancer response to Tumor Treating Fields (TTFields)

**DOI:** 10.1038/s41420-025-02615-5

**Published:** 2025-07-10

**Authors:** Kerem Wainer-Katsir, Adi Haber, Hila Fishman, Lianghao Ding, Michael D. Story, Renfei Du, Ulf D. Kahlert, Laura Mannarino, Federica Mirimao, Monica Lupi, Maurizio D’Incalci, Gitit Lavy-Shahaf, Hila Ene, Roni Frechtel-Gerzi, Zeina Drawshy, Antonia Martinez-Conde, Eyal Dor-On, Yaara Porat, Moshe Giladi, Uri Weinberg, Yoram Palti

**Affiliations:** 1 Novocure LTD, Haifa, Israel; 2 Department of Radiation Oncology, University of Texas Southwestern Medical Center, Dallas, TX USA; 3 Clinic for Neurosurgery, Heinrich-Heine University, Duesseldorf, Germany; 4 Chifeng Municipal Hospital, Chifeng, China; 5 Molecular and Experimental Surgery, University Clinic for General-, Visceral-, Vascular- and Trans-Plantation Surgery, Medical Faculty and University Hospital Magdeburg, Otto-von Guericke University, Magdeburg, Germany; 6 Laboratory of Cancer Pharmacology, IRCCS Humanitas Research Hospital, Milano, Italy; 7 Department of Biomedical Sciences, Humanitas University, Milano, Italy; 8 Department of Oncology, Istituto di Ricerche Farmacologiche Mario Negri IRCCS, Milano, Italy

**Keywords:** Cancer therapy, Cancer genomics

## Abstract

Tumor Treating Fields (TTFields) therapy is an approved cancer treatment modality, based on non-invasive application of electric fields to the tumor region. Proteomic and cell biology methods revealed a versatile mechanism of action to be involved in the response to TTFields. In the current research we performed whole transcriptome analysis across tumor types to identify pan-cancer responses to TTFields. For this we collected samples from control and TTFields-treated human cancer cell lines of gastric cancer, pancreatic cancer, ovarian cancer, non-small cell lung carcinoma, pleural mesothelioma, and glioblastoma. The transcriptomic analysis supported previous reported effects: downregulation of pathways associated with cell cycle, cell growth, and proliferation; downregulation of DNA replication and the FA-BRCA DNA repair pathway; and upregulation of cellular responses to stress—senescence, autophagy, and apoptosis. Notably, previously unrecognized downstream effects of TTFields were revealed on cellular metabolism, with downregulation of protein and RNA metabolism, and upregulation of steroid biosynthesis. Additional DNA repair pathways were also found to be downregulated, including nucleotide excision repair, base excision repair, and mismatch repair. In conclusion, this study revealed similar response patterns to TTFields across different tumor types, re-enforcing some already pinpointed mechanisms, while revealing new mechanisms. Unlocking these new mechanisms may allow identification of potential new cancer treatments for application together with TTFields based on mechanistical compatibility.

## Introduction

Tumor Treating Fields (TTFields) are electric fields that induce cancer cell death via disruption of critical cellular processes [1, 2]. TTFields therapy is FDA-approved for treatment of glioblastoma (GBM), pleural mesothelioma, and metastatic non-small cell lung carcinoma (NSCLC). Recently, the efficacy of TTFields therapy for treatment of NSCLC brain metastasis and pancreatic cancer has been demonstrated in phase 3 studies (NCT02831959 and NCT03377491) [3]. TTFields therapy is applied loco-regionally and non-invasively via arrays placed on the patient’s skin around the tumor area, that are connected to a portable, battery-operated electric field generator [4, 5].

The effects of TTFields—as demonstrated clinically and preclinically—are time and electric field intensity dependent, with longer treatment durations, higher usage per day, and higher intensities, being more effective [6–13]. The sensitivity of different cell lines varies in accordance with cell doubling time and genetic background, though specific mutational vulnerabilities have not yet been established [11, 14, 15]. Within the TTFields frequency range of 100–500 kHz, the optimal treatment frequency has been shown to be specific per the cancer type, for example, 200 kHz for GBM and ovarian cancer, and 150 kHz for NSCLC, pleural mesothelioma, pancreatic cancer, and gastric cancer [6–11, 15]. Normal cells have, however, been shown not to be affected by TTFields, attributed to the their different electrical properties relative to those of cancer cells [16].

TTFields may induce various types of cancer cell death. Exposure of cancer cells undergoing mitosis to TTFields leads to interference with normal mitotic spindle assembly and chromosome segregation, with subsequent mitotic arrest and possible mitotic cell death [4, 6, 7, 10, 14, 17, 18]. Most cells will however complete mitosis, giving rise to mainly abnormal progeny, characterized by aneuploidy, genome instability, ER stress, autophagy, and cytosolic micronuclei [14, 15, 19–21]. These cellular stresses may trigger a downstream anti-cancer immune response via activation of the inflammasome system or by induction of immunogenic cell death (ICD) [20–22]. Other known downstream effects associated with application of TTFields to cancer cells include induction of replication stress and impairment of the Fanconi Anemia (FA)-BRCA DNA damage repair (DDR) mechanism [4, 11, 18, 23–26]. These effects may eventually lead to accumulation of DNA damage and subsequent cell death.

Whole transcriptomics in response to TTFields have been performed in GBM [27–29], NSCLC [4, 18], and pleural mesothelioma [30] cell lines. In the current research we performed a meta-analysis of data generated from cells of various cancer types, some already published and some newly generated, to: broaden the understanding of cancer cell response to TTFields; examine the generality of the already recognized response pathways; and possibly identify new pan-cancer responses to TTFields.

## Results

### TTFields induce differential gene expression with a correlated response across cells from different tumor types

We performed transcriptomics analyses on control and TTFields-treated human cancer cell lines from different tumor types: gastric (AGS and KATOIII), pancreatic (AsPC1 and BxPC3), and ovarian (OVCAR3) cancer, collected by Novocure; NSCLC (A549, H1299, H157, H1650, and HCC4006), collected by Karanam et al. [18]; pleural mesothelioma (CD473 and CD60; patient-derived), collected by Mannarino et al. [30]; and GBM (GBM1, JHH520, NCH644, SF188; stem cells, grown as neurospheres), collected by Du and Kahlert. The cell lines used presented with different mutational backgrounds and different levels of driver mutations (Fig. S1): a low level for AsPC1 and BxPC3, a high level for AGS and H157, and an intermediate level for KATOIII, A549, H1299, HCC4006, OVCAR3, and H1650. For the other cell lines, complete genomic background was not available. The most frequently mutated genes were TP53, mutated in 11 out of the 16 tested cell lines, and CDKN2A, mutated in 8 of the cell lines. Most of the other mutations were prevalent in 5 or less cell lines, spread out between the different cell lines.

TTFields induced a decrease in cell count relative to control, with different magnitudes of response for different cell lines (Table 1). Cell lines responding strongly to TTFields (cell count of ≤50% after 48 h of TTFields exposure) could be found across the different cancer types, without distinguishing between cells with low (BxPC3), high (AGS, H157), or intermediate (A549, OVCAR3) mutational burden. Additionally, no specific mutation/s could be linked to the sensitivity to TTFields.

**Table 1:** Properties of the examined cell lines, TTFields treatment conditions, cellular cytotoxic response to TTFields, and the different transcriptomics methods employed for each dataset.

Tumor type (abbreviation)	Cell line	Tumor origin	Donor sex	Growth mode	Doubling time [h]	TTFields treatment					Transcriptomics method	Ref
						Frequency [kHz]	Intensity [V/cm RMS]	Duration [h]	Cell count [% of control]			
									24 h	48 h		
Gastric cancer (GasC)	AGS	Primary	Female	Adherent	32	150	1.7	24, 48	74	30	PolyA RNA-seq with Unique Molecular Identifiers (UMI)	^b^
	KATOIII	Metastatic	Male	Adherent	27				85	61		
Pancreatic cancer (PanC)	AsPC1	Primary	Female	Adherent	33	150			79	62		
	BxPC3	Metastatic	Female	Adherent	45				72	49		
Ovarian cancer (OvaC)	OVCAR3	Metastatic	Female	Adherent	54	200			64	41		
Non-small cell lung carcinoma (NSCLC)	A549	Primary	Male	Adherent	22	150 ^a^	1.5	12, 24, 48	88	47	Illumina Whole Genome HumanWG6 v4 Expression BeadChips	[18]
	H1299	Metastatic	Male	Adherent	20				83	75		
	H157	N/A	Male	Adherent	36				96	33		
	H1650	Metastatic	Male	Adherent	26				95	84		
	HCC4006	Metastatic	Male	Adherent	34				106	87		
Pleural Mesothelioma (Meso)	CD473	Primary	Male	Adherent	42	150	1.1	8, 24, 48	64	42	Whole RNA-seq	[30]
	CD60	Primary	Female	Adherent	44				73	73		
Glioblastoma (GBM)	GBM1	Primary	Male	Neurospheres	62	200	1.7	48	N/A	84	PolyA RNA-seq	^c^
	JHH520	Primary	Female	Neurospheres	56				N/A	71		
	NCH644	Primary	Female	Neurospheres	102				N/A	73		
	SF188	Primary	Male	Neurospheres	37				N/A	75		

^a^Not all were treated at the same frequency, specific details may be found in the original publication of the datasets.

^b^Conducted by Novocure LTD, Haifa, Israel.

^c^Conducted by Renfei Du and Ulf Kahlert.

**Fig. 1:**
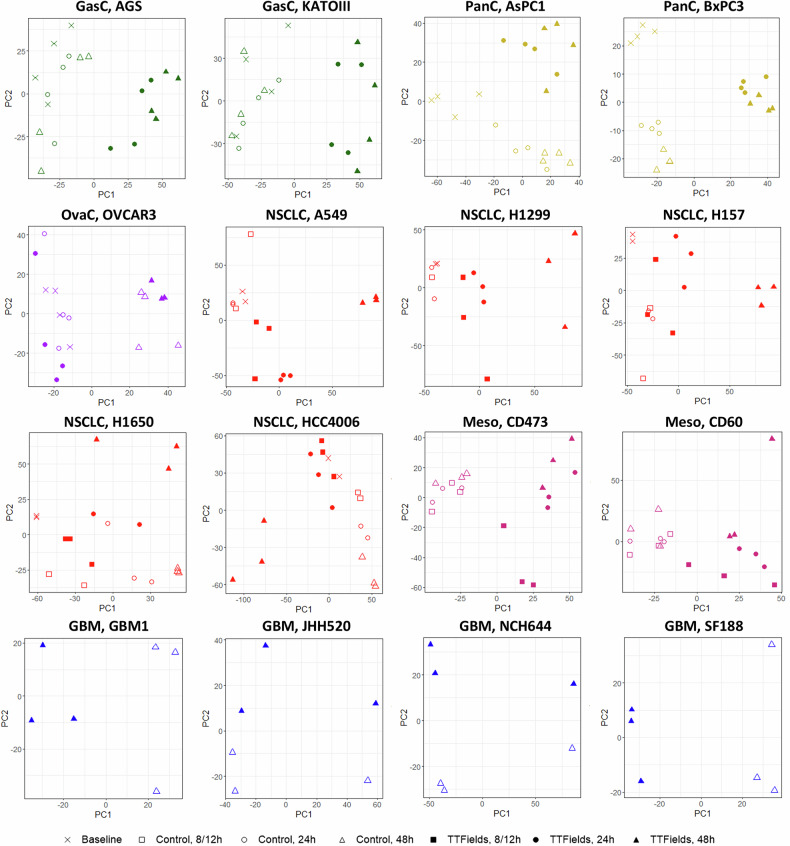
TTFields induce differential gene expression throughout tumor types. Principal components analysis (PCA) for the cell lines from the various tumor types (tumor type and cell line name within each panel, different colors for the different tumor types), showing TTFields-treated and control samples. Treatment times: 0 h (×), 8 or 12 h (□), 24 h (○), 48 h (∆). Treatment type: control (empty symbols), TTFields (filled symbols).

**Fig. 2:**
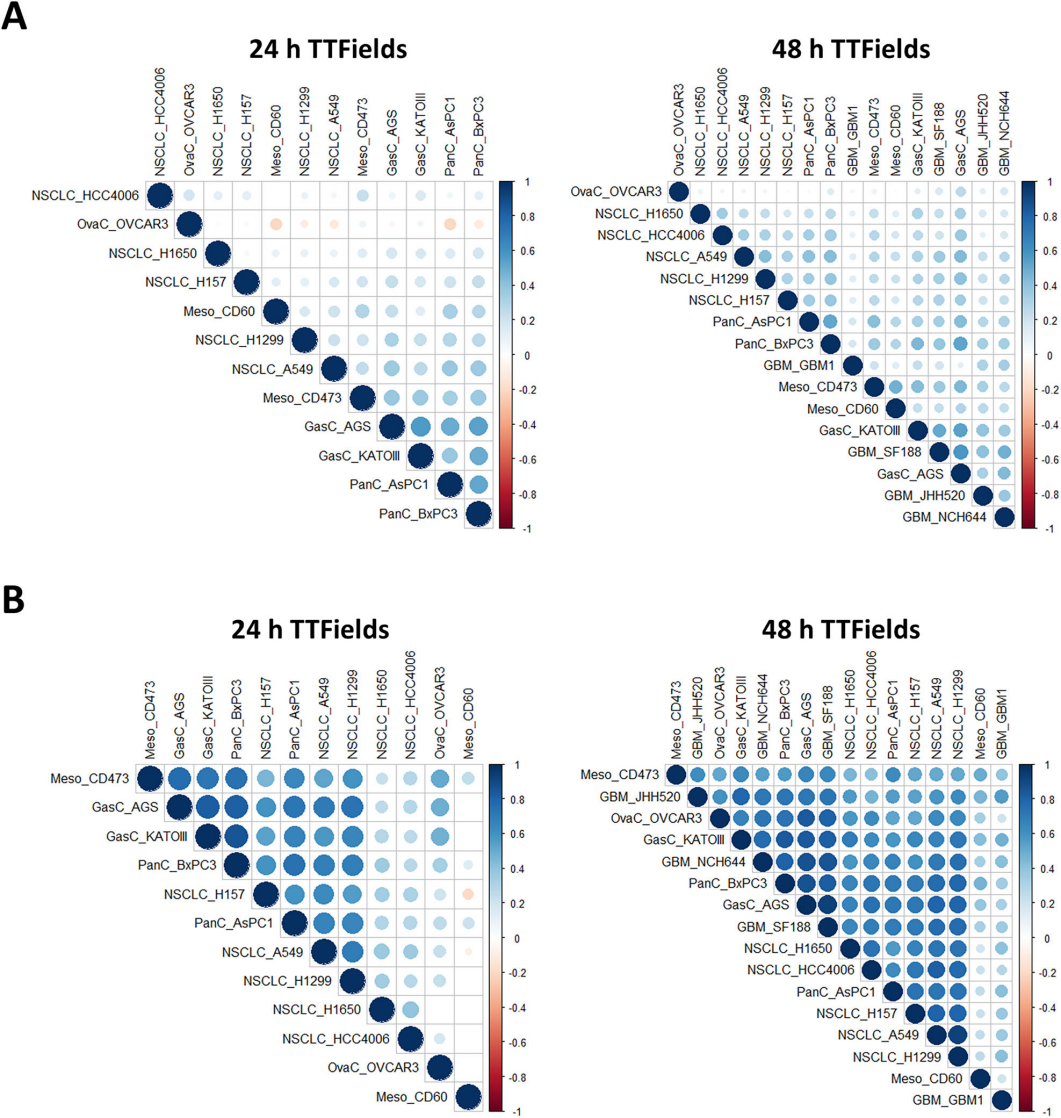
Response to TTFields shows correlation between cell lines from different tumor types. Spearman paired correlation analysis (*p* ≤ 0.05) between the various cell lines for gene expression’s log fold change (**A**) and Gene Set Enrichment Analysis’s (GSEA) Normalized Enrichment score (NES) according to the Reactome gene sets (**B**), following TTFields treatment of 24 or 48 h. Circle size corresponds to the level of correlation. Blue = positive correlation. Red = negative correlation.

Principal Components Analyses (PCA) showed, in most datasets, distinct differences between control and treated cells, differences that elevated following longer treatment durations (Fig. 1). We then analyzed differentially expressed genes in each of the datasets (most consistent genes are shown in Table S1). To examine similarities between the different cell lines in responses to TTFields, we performed Spearman paired correlation analysis of TTFields-treated versus control gene expression log fold change (logFC). This showed a weak positive correlation across most of the datasets at 24 and 48 h of TTFields treatment (Fig. 2A), with higher correlation seen for the latter.

We then performed Gene Set Enrichment Analysis (GSEA) according to the Reactome gene set. Spearman paired correlation of the Normalized Enrichment Scores (NES) revealed a positive correlation between the different datasets, which was higher than the correlation analysis at the gene expression level. Such correlations were evident after 24 h of TTFields treatment and were stronger at 48 h treatment (Fig. 2B).

### Common pathways are affected by TTFields across tumor types

**Fig. 3:**
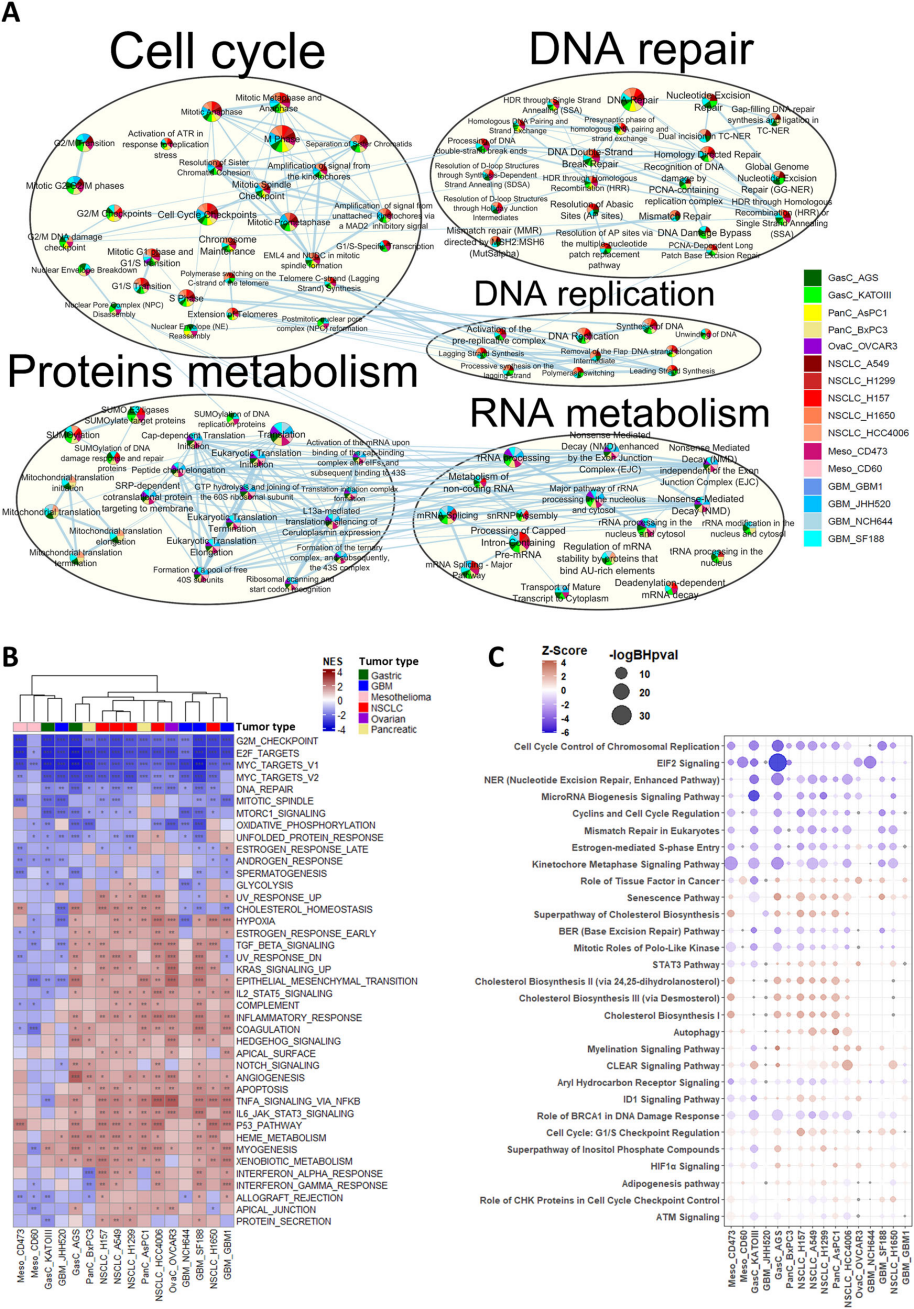
Common pathways are affected by TTFields across tumor types. **A** Enrichment map for 48-h TTFields-treatment datasets, representing commonly affected Reactome pathways. Analysis was done according to ActivePathways package, and the pathways clustered according to Reactome head pathways (header size indicative of the size of the cluster). Each node represents a pathway that was significantly altered in at least 5 datasets, with the pie colors depicting the different contributing datasets (see legend) and the pie size indicative of the common significance (Brown procedure). The edges correspond to the number of genes common to the pathways. The clustered circles were generated through AutoAnnotate. Only the top 5 pathways are shown. The complete map may be found in Fig. S2. **B** GSEA heatmap for 48-h TTFields-treatment datasets performed with MsigDB hallmark gene sets, showing pathways which were significant for at least 5 datasets. Normalized Enrichment Score (NES): Red = upregulated, Blue = downregulated. False Discovery Rate (FDR): * FDR ≤ 0.05, ** FDR ≤ 0.01, *** FDR ≤ 0.001. Cell lines from each tumor type are labeled with a different color. **C** Dot plot of IPA canonical pathways changed in response to TTFields application. A threshold *p*-value ≤ 0.05 and absolute log fold change ≥0.415 was applied. Only pathways that had a significance of BH *p*-value ≤ 0.05 in at least 5 datasets are shown. The circle color represents the Z-score. Red = upregulated, Blue = downregulated. The circle size corresponds to the -log BH *p*-value. The order of the datasets is according to the hierarchical clustering in (**B**).

We next used the ActivePathways package on the Reactome gene set to identify common pathways changed across datasets in response to TTFields (Table S2). We created an enrichment map to show significantly overlapping pathways and the number of datasets supporting each pathway for 48 h TTFields treatment, showing pathways common to at least 5 datasets (Fig. 3A for the top 5 pathways, and Fig. S2 for the complete map). This analysis identified pan-cancer changes; the largest clusters were those for cell cycle, DNA repair, and DNA replication. Other major clusters included protein and RNA metabolism.

We further performed GSEA, based on the MsigDB hallmarks gene set. To show the most significant results in one figure, we constructed a heatmap of the NES along with the False Discovery Rate (FDR), showing pathways common and significant for at least 5 datasets (Fig. 3B for 48 h treatment. Fig. S3 for all time points). The non-supervised hierarchical clustering of the cell lines showed that the response to TTFields was independent of tumor type. Clustering was also independent of the driver mutations of the various cell lines (Fig. S1). The hallmark gene sets associated with proliferation—MYC targets V1 and V2, E2F targets, G2M checkpoint, and mitotic spindle—were downregulated across all datasets; as was DNA repair. On the other hand, apoptosis, and p53 pathways were mainly upregulated throughout. Other upregulated pathways were associated with metabolism (mainly heme, cholesterol, and xenobiotics metabolism) and immune response (interferon α and ɣ response, inflammatory response, and more).

We next performed analysis based on the Ingenuity Pathway Analysis (IPA) canonical pathways to validate the results via a manually curated database. The dot plot shows the Z-score along with the −logBH *p*-value of the different pathways in the different datasets, showing pathways common and significant to at least 5 datasets (Fig. 3C for 48 h treatment). The pathways downregulated across all datasets belonged to signaling pathway categories of: (1) cell cycle regulation; (2) cellular growth, proliferation and development; and (3) cellular response and injury (Table S3). Per the latter category, DNA replication, recombination and repair was identified as a top function for pathways that were downregulated, including nucleotide excision repair (NER), base excision repair (BER), mismatch repair (MMR), and role of BRCA1 in DNA damage response. However, pathways associated with cellular stress and injury without this top function were upregulated—senescence, autophagy, and Coordinated Lysosomal Expression and Regulation (CLEAR) signaling. Consistent elevations were also seen in steroid biosynthesis from the fatty acids and lipids biosynthesis category (namely cholesterol biosynthesis I, II, and III, and superfamily of cholesterol biosynthesis), whereas pathways associated with top functions of gene expression and protein synthesis (eIF2 and microRNA biogenesis signaling) were downregulated.

**Fig. 4:**
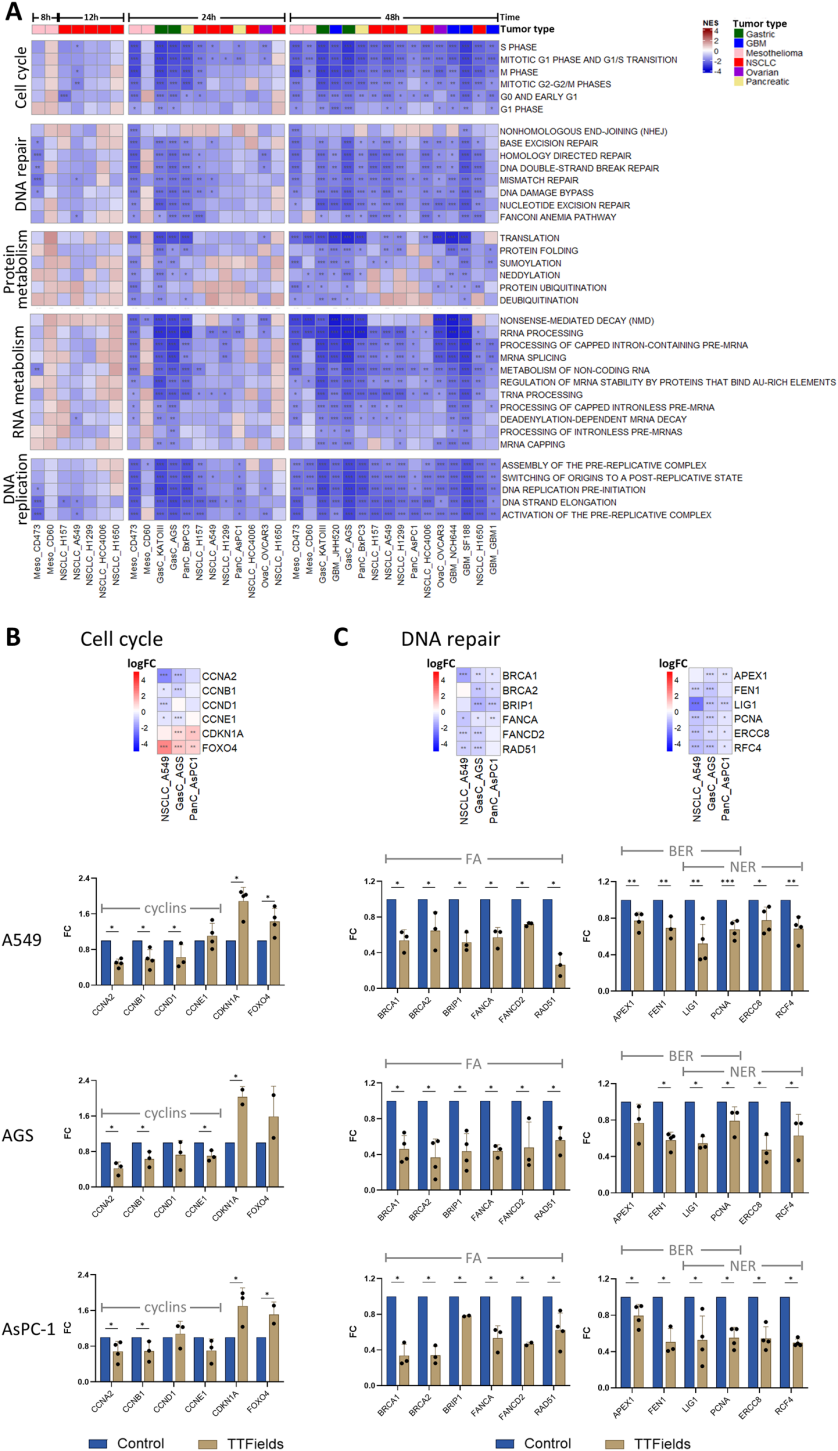
A closer look at the common responses to TTFields. **A** Heatmaps across TTFields-treatment time points of GSEA performed with Reactome pathways. Shown are common head pathways that had at least 10 sub-pathways changed across at least 5 datasets following TTFields application (as determined in Fig. 3A): Cell cycle; DNA repair; protein metabolism; RNA metabolism; and DNA replication. Normalized Enrichment Score (NES): Red = upregulated, Blue = downregulated. Cell lines from each tumor type are labeled with a different color. The order of the datasets is according to the hierarchical clustering in Fig. 3B. Heat maps for expression of specific genes based on the RNA-seq data, and qPCR data for expression levels of these genes, in A549, AGS, and AsPC1 cells treated with TTFields for 48 h versus control samples. Selected genes are pivotal for cell cycle (**B**) and for DNA repair pathways: FA-BRCA, BER, and NER (**C**). For heatmaps log fold change (logFC): Red = upregulated, Blue = downregulated; *p ≤ 0.05, **p ≤ 0.01, ***p ≤ 0.001. For qPCR results represent the mean ± SEM; *p ≤ 0.05, **p ≤ 0.01, and ***p ≤ 0.001, calculated using two-sided multiple *t*-tests.

We next examined changes over time of common pathways that responded to TTFields. We focused on the most affected pathways, those that following TTFields application had at least 10 sub-pathways changed across at least 5 datasets (Fig. 3A). For these pathways we created Reactome pathway-based GSEA heat maps broken down into their major sub-pathways (Fig. 4A). This analysis showed, in cells of all tumor types, significant downregulation of all cell cycle phases after 24 h of TTFields application, and more so after 48 h of treatment, with G0/early G1 downregulated to the lowest extent. The same time-dependent downregulation was seen across tumor types for most DNA repair pathways, including BER, NER, MMR, and the FA-BRCA pathway. The non-homologous end joining (NHEJ) pathway was, however, not affected by TTFields application at any of the time points. Changes in protein and RNA metabolism were evident mainly following 48 h of TTFields. The sub-pathway breakdown showed translation, SUMOylation, and folding to be the major protein metabolism pathways affected by TTFields; and that most RNA metabolism pathways are affected. The same time-dependent response seen for cell cycle and DNA repair of downregulation already after 24 h of treatment was also evident for DNA replication-related pathways, with all sub-pathways affected.

**Fig. 5:**
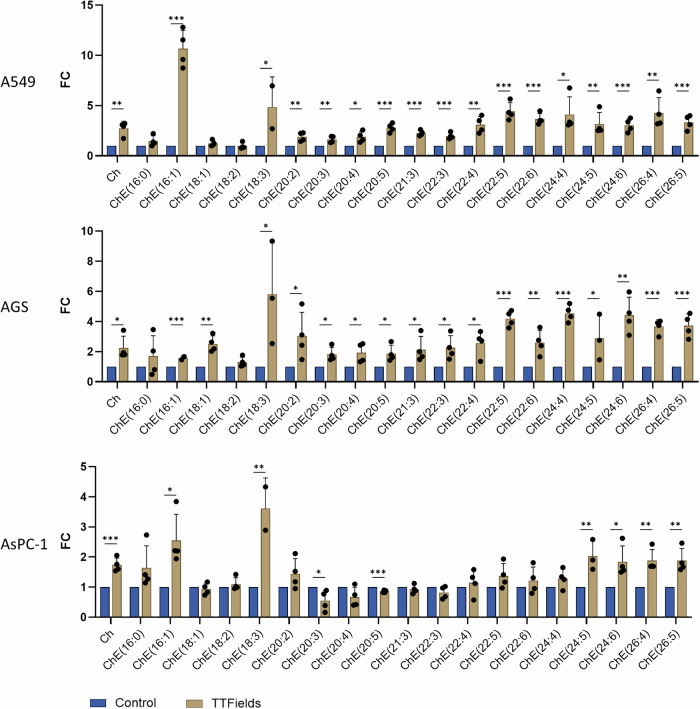
Validation of the cholesterol homeostasis response to TTFields. Cholesterol and cholesterol ester (CE) levels in A549, AGS, and AsPC1 cells treated with TTFields for 48 h versus control samples. Results represent the mean ± SEM; *p ≤ 0.05, **p ≤ 0.01, and ***p ≤ 0.001, calculated using two-sided multiple *t*-tests.

To validate the results, we selected 3 cell lines of different tumor types, that presented different sensitivities to TTFields and various mutational burden: lung A549 cells—low TTFields sensitivity, intermediate mutational burden (wild type TP53); gastric AGS cells—high TTFields sensitivity, high mutational burden (wild type TP53); and pancreatic AsPC1 cells—low TTFields sensitivity, low mutational burden (mutated TP53). We used qPCR to examine effects of 48-h treatment with TTFields on cell cycle (Fig. 4B) and DNA repair pathways (Fig. 4C). This examination showed significantly decreased expression of several cyclins across the tested cell lines. On the other hand, the DNA damage-triggered cyclin dependent kinase (CDK) inhibitor CDKN1A (p21) was significantly upregulated; along with FOXO4, which transcriptionally activates CDKN1A. Examined genes involved in DNA repair pathways, including the FA-BRCA pathway, and the BER and NER pathways, were all downregulated across the three cell lines. We used lipidomics to examine the effects of 48-h TTFields treatment on cholesterol homeostasis. This examination showed significant elevation of cellular cholesterol levels, as well as of most of the cholesterol esters (Fig. 5). The most affected cholesterol ester across the tested cell lines was CE 18:3 (linolenate-cholesterol ester), elevated fourfolds throughout.

### The response to TTFields resembled that of known cascades of upstream transcriptional regulators and shared similarities with responses to various anticancer drugs

**Fig. 6:**
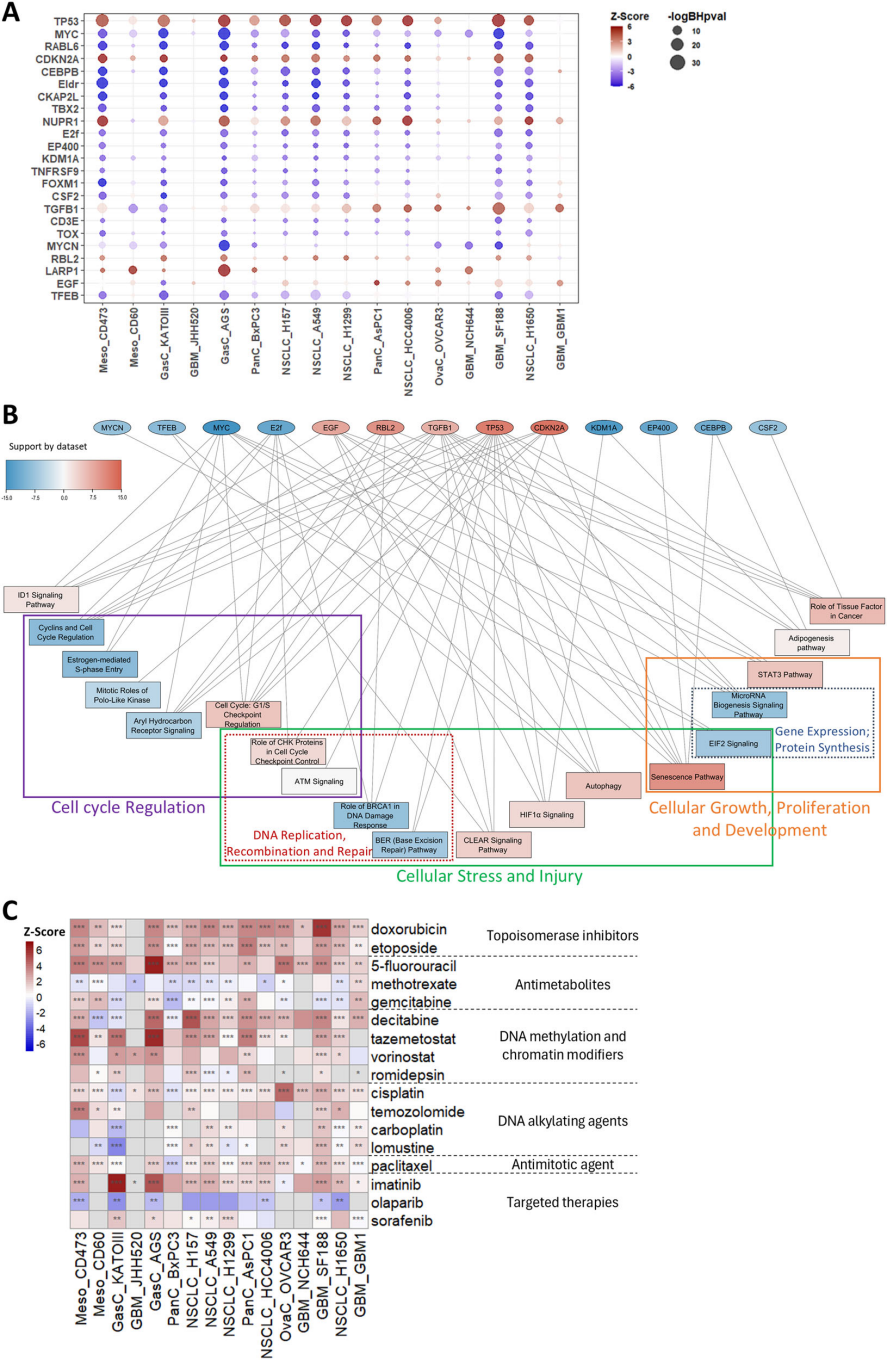
Cascades of upstream transcriptional regulators, network analysis in response to TTFields, and drug similarity analysis. **A** Dot plot of the cascade of upstream transcriptional regulators enriched following TTFields application according to IPA. The circle color represents the Z-score. Red = upregulated, Blue = downregulated. The circle size corresponds to the -log BH *p*-value. Only upstream regulators that had a significance of BH *p*-value ≤ 0.01 and Z-score ≥4 or ≤ -4 in at least 5 datasets are shown. The order of the datasets is according to the hierarchical clustering in Fig. 3B. **B** Network analysis of relationships between upstream regulators (from **A**, shown as ellipses) and canonical pathways (from Fig. 3C, shown as rectangles) changed following application of TTFields. The shape color, red or blue, and tone intensity indicate a score representing how many datasets favored up- or down-regulation of each entity, respectively. Canonical pathways are clustered according to the most common pathway categories: cell cycle regulation; cellular stress and injury; and cellular growth, proliferation and development. Canonical pathways are further clustered according to most common top functions: DNA replication, recombination and repair; gene expression; and protein synthesis. **C** Heatmap of drugs as IPA upstream regulators compared to TTFields. The drugs are arranged according to their mechanisms of action. Z-score: Red = upregulated, Blue = downregulated. * BH *p*-value ≤ 0.05, ** BH *p*-value ≤ 0.01, *** BH *p*-value ≤ 0.001.

We performed the IPA upstream regulators analysis to identify transcriptional cascades involved in the response to TTFields (Fig. 6A). The most significantly changed pathways across at least 5 datasets included those downstream to tumor suppressor genes, such as TP53 and CDKN2A, which were upregulated, and those downstream to oncogenes, including MYC and E2F, which were downregulated. For Myc, the expression of the gene itself was downregulated in many of the datasets (Fig. S4A). However, changes in a cascade associated with an upstream regulator do not necessarily imply changes in expression levels of the upstream regulator itself, as may be appreciated by the fact that TP53 and CDKN2A were mutated in most cells (Fig. S1), yet their downstream pathways were still among the most significantly changed following TTFields application (Fig. 6A).

Examination of the cellular role of the identified upstream regulators revealed that most are associated with proliferation, apoptosis, and growth; and many are involved in cell cycle progression and the various cell cycle phases (Table S4). Interestingly, exploration of proteins directly regulated by these upstream regulators identified CDKN1A as the most common, subjected to the regulation of 8 of these upstream regulators. The expression data showed that this gene was elevated by about twofold in most of the datasets (Fig. S4B for complete RNA-seq based data, Fig. 4 B for qPCR data of 3 selected cell lines).

A network analysis, demonstrating interconnections between the canonical pathways and the upstream regulators identified in the cellular response to TTFields, is shown in Fig. 6B. This network also highlights up- and down-regulated pathways according to their category and top function: (1) within the cell cycle regulation category most pathways are downregulated, except for those associated with checkpoints regulation; (2) within the cellular growth, proliferation and development category, pathways associated with gene expression and protein synthesis are downregulated, while other pathways are upregulated; and (3) within the cellular response and injury category most pathways are upregulated, excluding those associated with DNA replication, recombination and repair that are not included within the cell cycle regulation category.

Finally, we performed IPA drug analysis to identify similarities between responses of cancer cells to TTFields and to that of anticancer drugs of various mechanisms of action (Fig. 6C). TTFields demonstrated high correlation with the antimitotic agent paclitaxel, and with various DNA damaging agents—some topoisomerase inhibitors, antimetabolites, DNA methylation and chromatin modifiers, and DNA alkylating agents. Interestingly, from the group of targeted therapies, response to TTFields was the opposite of the response to the poly (ADP-ribose) polymerase (PARP) inhibitor, olaparib.

## Discussion

This work presents an analysis of the transcriptomic response to TTFields of cancer cells from various tumor types. The cells were characterized by different cellular sensitivities to TTFields, as expected by the different doubling times. Nevertheless, there were distinct transcriptomic changes for cells treated with TTFields relative to untreated cells across tumor types, suggesting that these were pan-cancer responses. A connection between the transcriptomic changes and specific driver mutations could not be established. While previous protein-based tumor-type-specific studies have shown various aspects of TTFields mechanism [1, 2], this work is the first to demonstrate whole-transcriptome pan-cancer analysis, with a consistent mechanism of action throughout different tumor types. This study also demonstrates that TTFields do not act by one distinct mechanism of action, but rather in several different fronts, to interfere with cellular processes pivotal for cancer cell proliferation.

While transcriptomic analysis could not directly provide evidence for the antimitotic effect of TTFields, as changes associated with such an effect are physical and not observed at the mRNA level, a clear and consistent downregulation of cell cycle, cell growth, and proliferation was evident throughout all datasets. qPCR examinations validated these results, demonstrating downregulation of several different cyclins, most of them not identified before to be altered following TTFields application. The similarity between the response to TTFields and the response to the antimitotic drug paclitaxel serves as an additional indirect indication for the antimitotic effect of TTFields.

Such cell cycle arrest could be the direct consequence of the antimitotic effect, or alternatively a downstream cellular response to injury. Interestingly, the p21 encoding gene, CDKN1A, previously shown to be upregulated by TTFields at the protein level [11, 26, 31], was also identified in the transcriptomics data to be upregulated following TTFields application. qPCR examinations validated the elevation of CDKN1A mRNA levels, and also demonstrated increased expression of FOXO4, which transcriptionally activates CDKN1A. These two genes are involved in the induction of cell cycle arrest in response to DNA damage [32, 33], suggesting the induction of DNA damage following TTFields application.

DNA damage is a type of cellular injury previously shown to be associated with TTFields treatment, as a direct consequence of replication stress induced by TTFields, or an indirect outcome associated with TTFields-induced downregulation of DDR pathways [4, 11, 18, 23, 25, 26]. The Reactome- and IPA-based transcriptomic analysis supported the previously seen TTFields-induced downregulation of DNA replication and the FA-BRCA repair pathway; the latter validated by qPCR analysis. The current study revealed for the first time that other DDR pathways are affected by TTFields application, including NER, BER, and MMR. The downregulation effect on NER and BER were further validated by qPCR.

Such a global effect of TTFields on DDR may have major implications, suggesting potential for synthetic lethality upon concurrent treatment of TTFields with anticancer drugs that are DDR inhibitors. For example, preclinical efficacy of TTFields together with PARP or ataxia telangiectasia and Rad3-related protein (ATR) inhibitors, has been demonstrated [4, 26, 34]. Interestingly, we show that while TTFields share mechanistical characteristics with many anticancer drugs, the mechanism of TTFields is quite the opposite of that of the PARP inhibitor olaparib. This result supports the high synergy demonstrated for the application of TTFields together with PARP inhibitors [26]. As no effect was seen for TTFields on the NHEJ pathway, co-application with inhibitors of this DNA repair pathway may potentially also provide synergy.

The effect of TTFields on DDR pathways may also indicate the induction of cellular conditional vulnerability to DNA damaging agents. This concept is already exploited clinically in the approved indications for TTFields therapy, together with the DNA alkylating agent temozolomide for treatment of GBM, and together with the DNA intercalating agent cisplatin for treatment of pleural mesothelioma [5]; and effectiveness concomitant with radiation therapy is currently under clinical investigation (TRIDENT study, NCT04471844).

MsigDB and IPA showed that certain cellular responses to injury were upregulated following the application of TTFields, those that serve as cellular defense or self-destruct mechanisms. This included senescence, autophagy, and apoptosis, previously shown in cellular assays to be induced by TTFields application [14, 15, 19, 20]. Activation of these mechanisms suggests the involvement of ER stress, mitochondrial (oxidative) stress, and/or DNA damage in the response to TTFields. IPA also pinpointed the CLEAR pathway as part of the response to TTFields. The CLEAR pathway regulates lysosomal biogenesis and function, and was recently suggested to be involved in regulation of additional lysosome-associated processes, including autophagy, endocytosis, phagocytosis, and immune response [35]. Future studies may examine the importance of the CLEAR pathway in the response to TTFields.

Metabolism was also found to be affected downstream of TTFields application, with Reactome and IPA showing downregulation of protein and RNA metabolism, while MsigDB and IPA showing upregulation of steroid biosynthesis. The latter was validated by lipidomics analysis, showing elevations of cholesterol and many of the cellular cholesterol esters (the means for storing excess cholesterol for avoiding cholesterol toxicity). While the effects on cell cycle and DNA damage were evident already at early timepoints, metabolic alternations appeared only following 48 h treatment. This is the first time effects of TTFields on metabolism have been described, requiring further investigation to understand the mechanism and the extent of these responses.

Altered lipid metabolism is one of the hallmarks of cancer [36, 37]. Cancer cells use cholesterol as an energy source and as a membranal structural component when rapidly dividing. Cholesterol also serves as an important lipid messenger for signal transduction, activating oncogenic signals and inhibiting apoptosis. Furthermore, alternations of lipid metabolism have been linked to the development of tumor drug resistance. Overall, the upregulation of cholesterol metabolism may be a defense mechanism of cancer cells against TTFields. Reduced cancer cell sensitivity to TTFields has recently been described to develop via the activation of the PI3K/AKT pathway [38], a pathway which is involved in regulating cholesterol homeostasis. That work suggested the use of PI3K inhibitors, such as alpelisib, for augmenting the effects of TTFields. With the results depicted here regarding the involvement of cholesterol and cholesterol esters in the response to TTFields, the possibility of boosting TTFields activity by co-application with inhibitors of cholesterol biosynthesis (i.e. statins) or cholesterol esterification warrants examination.

An effect on the immune response was evident from the MsigDB dataset, however, could not be identified by the other databases. Importantly, studies on isolated cancer cells are performed in a controlled and standardized environment, with lack of dynamic tissue interplay and no involvement of immune cells, hence are limited in their ability to identify immunological cues. Nevertheless, specific cell biology assays have previously shown ICD induction and inflammasome activation in cancer cells in response to TTFields; and the benefit of co-applying TTFields with immune checkpoint inhibitors was demonstrated in vivo [20, 22] and in the clinic [39].

While the high-throughput screen described herein sheds light on the response of cancer cells to TTFields, it should be kept in mind that there are certain limits to in silico transcriptomic analysis. Since transcriptomic analysis examines only mRNA expression levels, additional relevant mechanisms may be overlooked if they involve post-translational modification, include protein-protein interactions, depend on cellular localization, etc. As mentioned, the antimitotic effect of TTFields falls within this group.

To conclude, the integration of our findings shows that the transcriptomic fingerprint of TTFields corresponds to many cancer-related pathways. While most anticancer agents trigger a specific response, TTFields as a single modality was able to recapitulate the mechanism of various drugs. As cancer cells develop resistance to treatment, the use of drug combination is a common practice in cancer therapy; however, combinations of such systemic drugs, each having its own side effects, take a tall from the patient. Having a locoregional, low toxicity treatment act by a multitude of mechanisms offers benefit in this respect. The new responses to TTFields revealed in our study on DDR pathways previously unstudied in the context of TTFields and on various aspects of metabolism provide avenues for extending TTFields research, and for exploring-based on mechanistic compatibility-co-application strategies with drugs for potentially increasing treatment efficacy of TTFields.

## Materials and methods

### Cell lines

Gastric AGS (RRID: CVCL_0139) and KATOIII (RRID: CVCL_0371), pancreatic AsPC1 (RRID: CVCL_0152) and BxPC3 (RRID: CVCL_0186), ovarian OVCAR3 (RRID: CVCL_0465), and lung A549 (RRID: CVCL_0023) human cell lines were obtained from the American Tissue Culture Collection (ATCC) between 2011 and 2021. AsPC1 and BxPC3 cell lines were authenticated using short tandem repeat (STR) profiling. Cells were grown in a 37 °C humidified incubator supplied with 5% CO_2_ with the following media: AGS and A549—F12K media supplemented with 10% fetal bovine serum (FBS); KATOIII—IMDM media supplemented with 20% FBS; OVCAR3—RPMI1640 media supplemented with 20% FBS; AsPC1 and BxPC3—RPMI1640 media supplemented with 10% FBS. All media were further supplemented with 2 mM L-glutamine and 50 μg/ml penicillin/streptomycin. F12K and IMDM media were purchased from Biological Industries (Beit Haemek). RPMI1640, FBS, and supplements were purchased from (Gibco). Cells were routinely examined for mycoplasma contamination.

GBM1 cells (RRID: CVCL_DG57) were kindly provided by A. Vescovi (San Raffaele Hospital, Milano, Italy). NCH644 cells (RRID: CVCL_X914) were kindly provided by C. Herold-Mende (Heidelberg University, Heidelberg, Germany). SF188 cells (RRID: CVCL_6948) were kindly provided by E. Raabe (Johns Hopkins, Baltimore, MA, USA). JHH520 cells (RRID: CVCL_VT33) were kindly provided by G. Riggins (Johns Hopkins, Baltimore, MD, USA). Cell lines were authenticated using STR profiling. The cells were cultured in DMEM media (Thermo Fisher) supplemented with 30% F-12 (Thermo Fisher), 2% B-27 (Thermo Fisher), 0.1% human EGF (Peprotech, AF-100-15), 0.1% FGF (Peprotech, 100-18B), 0.1% Heparin (Sigma, H0878), and 50 μg/ml penicillin/streptomycin (Sigma Aldrich) to spontaneously form spheroids [40].

### Cell line mutational status

A549, AGS, AsPC1, BxPC3, HCC4006, KATOIII, H1299, H1650 and OVCAR3 mutations were downloaded from CellModelPassport [41]. The table of mutation consisted of both SNVs and CNVs. The definitions of the CNVs were taken from https://depmap.sanger.ac.uk/documentation/datasets/copy-number/. SNV and CNV tables for H157 were downloaded from Depmap [42]. Mutations were filtered using R and Rstudio [43, 44] to include only those that were in driver genes. The list of genes was screened in Depmap table for mutations in H157. For the other cell lines, only the mutations screened and provided in the manuscripts were available [30, 40, 45, 46]. Results for alterations that were common to at least 2 cell lines were collected and are reported as an oncoprint [47].

### TTFields application

TTFields were applied to cells using the inovitro^TM^ system as previously described [6, 7, 14], using TTFields treatment frequency, intensity, and duration as specified in Table 1. Cell count was determined using flow cytometry or manual count and expressed as a percentage relative to control.

### RNA purification, library preparation, and sequencing

#### Gastric/Pancreatic/Ovarian cancer dataset

RNA was purified with TRI reagent as previously described [48] from 4 independent repeats, and quantified with Nanodrop, Qubit (Thermo Fisher Scientific) and TapeStation (Agilent). For cDNA libraries preparation, the polyA fraction (mRNA) was purified, followed by fragmentation and generation of double-stranded cDNA. Agencourt AMPure XP beads (Beckman Coulter) were used for cleanup, followed by end repair, UMIs addition, adapter ligation and PCR amplification steps. Sequencing was done on Illumina NovoSeq 6000 system. Library generation and sequencing were done by the Crown Genomics institute of the Nancy and Stephen Grand Israel National Center for Personalized Medicine, Weizmann Institute of Science.

#### GBM dataset

RNA was purified with TRI reagent as previously described [48] from 3 independent repeats, quantified using Nanodrop and Qubit RNA HS Assay (Thermo Fisher Scientific), and quality measured by capillary electrophoresis using the FragmentAnalyzer and the DNF-471 Standard Sensitivity Assay (Agilent Technologies Inc., Santa Clara, USA). For cDNA library, preparation was performed by VAHTS Universal V6 RNA-seq Library Prep Kit (Illumina) according to the manufacturer’s protocol, including mRNA capturing, fragmentation, cDNA synthesis, adapter ligation and library amplification. Bead purified libraries were normalized and sequenced on the HiSeq3000 system (Illumina). Library generation and sequencing were performed by the Biologisch-Medizinisches Forschungszentrum (BMFZ) Facility.

#### Pleural mesothelioma and NSCLC datasets

Cell cultures and transcriptomic methods were described previously ([30] for pleural mesothelioma [18], for NSCLC).

### RNA quantification and differential expression

For RNA-seq samples (all but the NSCLC dataset), Fastq files were quality control screened with FASTQC [49]. Reads were aligned to the GENCODE human reference GRCh38.p13 with annotation file gencode.v36.annotation.gtf (both downloaded from https://www.gencodegenes.org/human/) using STAR-2.7.6a [50]. Alignment and FastQC were assessed using multiqc [51]. Bam files were indexed using samtools [52]. Output files were organized into a sample/gene matrix. Normalization of reads into log Counts Per Million (CPM) was done by edgeR [53].

For the gastric/pancreatic/ovarian cancer dataset, a pre-alignment step was taken, where UMIs from R2 were converted to the header using UMI_tools [54]. Following STAR alignment and SAMtools indexing [52], files were deduplicated using UMI_tools. Reads per gene were counted with HTseq [55].

For the NSCLC dataset, normalized gene expression was used as provided in ref. [18]. The probe with the highest expression across all samples was taken as the representative probe per gene.

For the RNA-seq samples, ComBat-seq [56] was used to remove batch effect, and Limma-voom [57] was used for processing. In all datasets, outliers were screened using arrayQualityMetrics [58], and samples that failed 2/3 tests were excluded as outliers. Differential expression was made for every TTFields treatment time point against its control using Limma [59].

### Data analysis and visualizations

Data analysis and visualizations were performed using R [43] and Rstudio [44], unless specified otherwise. Packages for visualizations included pheatmap [60], ComplexHeatmap [47], ggplot2 [61], heatmaply [62], seriation [63], and dendextend [64]. Table and string organization were done using dplyr [65], tidyverse [66], tidyR [67], and stringr [68]. Genes annotations were done with STRINGdb [69].

### Gene set enrichment analysis

GSEA was performed on the gene lists preordered by their t score, using the GSEA software [70] against the Hallmark genes set by the broad MsigDB [70] and against the Reactome gene set [71].

### Datasets Spearman paired correlation analysis

Spearman correlation between logFC and Normalized Gene set score from the various datasets was preformed according to the pairwise complete observations method, and visualized using corrplot [72].

### Active Pathways and enrichment map

*P*-values of the differential expression analysis were taken into ActivePathways [73], that calculated the Brown common *p*-value. The cutoffs for the significance were the default of the package, with a gene list size of between 10 and 500 genes and a Brown adjusted *p*-value of ≤0.05. Output was then, through Cytoscape [74], visualized into an enrichment map [75] for common significance pathways according to cell line contribution to each pathway. The pathways were clustered according to the Reactome mother node classification using AutoAnnotate [76].

### Canonical pathways, upstream regulators analysis and drug analysis

Ingenuity canonical pathways and upstream regulators analysis (including drug analysis) were done using QIAGEN Ingenuity Pathway Analysis (IPA, https://digitalinsights.qiagen.com/IPA, summer update 2023) [77]. For the differential genes the cutoffs included a basic expression (AveExp) of 1 (for NSCLC Intensity of 1, and for RNA-seq log2 of CPM of 1). The fold change was with a change of at least 25% and the *p*-value at most 0.05. Mechanism of action of the drugs was taken from https://clue.io/data/CMap2020#LINCS2020/compoundinfo_beta.txt.

### Network analysis

The build option of IPA (QIAGEN Inc., https://digitalinsights.qiagen.com/IPA) was used to connect between the upstream regulators and the canonical pathways with the highest Z-scores and BH *p*-values. The network was extracted and further analyzed and visualized with Cytoscape. Only connected upstream regulators and canonical pathways were kept for the representation. R was used to sum upstream regulators and canonical pathways with BH *p*-value ≤ 0.05 and Z-score ≥0.1 within the different datasets, such that datasets in which an entity was upregulated were assigned a value of + 1 and those in which an entity was downregulated were assigned a value of -1.

### Quantitative polymerase chain reaction (qPCR)

Total RNA was isolated from the cells using the Total RNA Purification Plus Kit (17200, Norgen Biotek) and cDNA was synthesized (95047, Quantabio) according to the manufacturer’s instructions. Quantitative real-time PCR was carried out with the fast SYBR green master mix (4385614, Applied Biosystems) on a QuantaStudio 1 thermocycler (Applied Biosystems, Singapore), utilizing the primers listed in Table 2. The Housekeeping genes *UBC* and *PSMB4* were used for normalization using the QuantaStudio software.

**Table 2:** List of primers for qPCR.

Gene	Forward	Reverse
CCNA2	CGCTGGCGGTACTGAAGTC	GAGGAACGGTGACATGCTCAT
CCNB1	AATAAGGCGAAGATCAACATGGC	TTTGTTACCAATGTCCCCAAGAG
CCND1	GCTGCGAAGTGGAAACCATC	CCTCCTTCTGCACACATTTGAA
CCNE1	GCCAGCCTTGGGACAATAATG	CTTGCACGTTGAGTTTGGGT
CDKN1A	TGTCCGTCAGAACCCATGC	AAAGTCGAAGTTCCATCGCTC
FOXO4	GGCTGCCGCGATCATAGAC	GGCTGGTTAGCGATCTCTGG
BRCA1	ACCTTGGAACTGTGAGAACTCT	TCTTGATCTCCCACACTGCAATA
BRCA2	CACCCACCCTTAGTTCTACTGT	CCAATGTGGTCTTTGCAGCTAT
BRIP1	CTTACCCGTCACAGCTTGCTA	CACTAAGAGATTGTTGCCATGCT
FANCA	TTTGCTTGAGGTAGAAGGTCCA	CCCGGCTGAGAGAATACCCA
FANCD2	AAAACGGGAGAGAGTCAGAATCA	ACGCTCACAAGACAAAAGGCA
RAD51	CAACCCATTTCACGGTTAGAGC	TTCTTTGGCGCATAGGCAACA
APEX1	CAATACTGGTCAGCTCCTTCG	TGCCGTAAGAAACTTTGAGTGG
FEN1	ATGACATCAAGAGCTACTTTGGC	GGCGAACAGCAATCAGGAACT
LIG1	GCCCTGCTAAAGGCCAGAAG	CATGGGAGAGGTGTCAGAGAG
PCNA	GCGTGAACCTCACCAGTATGT	TCTTCGGCCCTTAGTGTAATGAT
ERCC8	ATGCTGGGGTTTTTGTCCG	TCTCCGTGTTGACTCTGCTCT
RCF4	CCGCTGACCAAGGATCGAG	AGGGAACGGGTTTGGCTTTC
UBC	CTGGAAGATGGTCGTACCCTG	GGTCTTGCCAGTGAGTGTCT
PSMB4	GAAGCGTTTTTGGGGTCGC	GAGTGGACGGAATGCGGTA

### LC–MS for lipidomics analysis

Three hundred microliters of a lipid extraction solvent consisting of 1:1 methanol:butanol were added to ~2 × 10⁶ cell pellets, vortexed for 20 min and centrifuged at 18,000 × *g* for 15 min at 4 °C. The cleared supernatants were collected, and 2 µL injected for the LC-MS analysis using a Vanquish Flex HPLC system coupled to an Orbitrap Exploris 240 mass spectrometer and a Acclaim C30 column (Thermo Fisher Scientific). The mobile phases consisted of 60:40 acetonitrile:water with 10 mM ammonium formate and 0.1% formic acid (A) and 90:10 isopropanol:acetonitrile with 10 mM ammonium formate and 0.1% formic acid (B), and the gradient was programmed as follows: 0–3 min 30% B, 3–5 min 43% B, 5–5.1 min 55% B, 5.1–8 min 60% B, 8–18 min 65% B, 18–24 min 85% B, 24–26 min 100% B, and 26–33 min 30% B. The Orbitrap Exploris 240 operated in both positive and negative ionization modes across a mass range of 120–1700 m/z. Lipid identification was performed using LipidSearch 5.0 (Thermo Fisher Scientific), and normalization of lipid peak areas was performed based on cells count for each sample. Quality control included the use of UltimateSPLASH™ ONE (Avanti, #330820) and lipid-labeled standards from various classes to ensure comprehensive coverage.

## Supplementary information

Supplementary figures

Table S1

Table S2

Table S3

Table S4

## Data Availability

The original RNA seq data generated for this study have been deposited in the European Nucleotide Archive (ENA) at EMBL-EBI under accession number PRJEB87601. Further inquiries can be directed to the corresponding author.
