# Supplementary material for: The transcriptomic fingerprint of cancer response to Tumor Treating Fields (TTFields)
Source: Cell Death Discov. 2025 Jul 10;11:319. doi: 10.1038/s41420-025-02615-5 (PMC12246047; doi:10.1038/s41420-025-02615-5)
Supplement: Supplementary file 1 — Supplementary figures [file 41420_2025_2615_MOESM1_ESM.pdf]

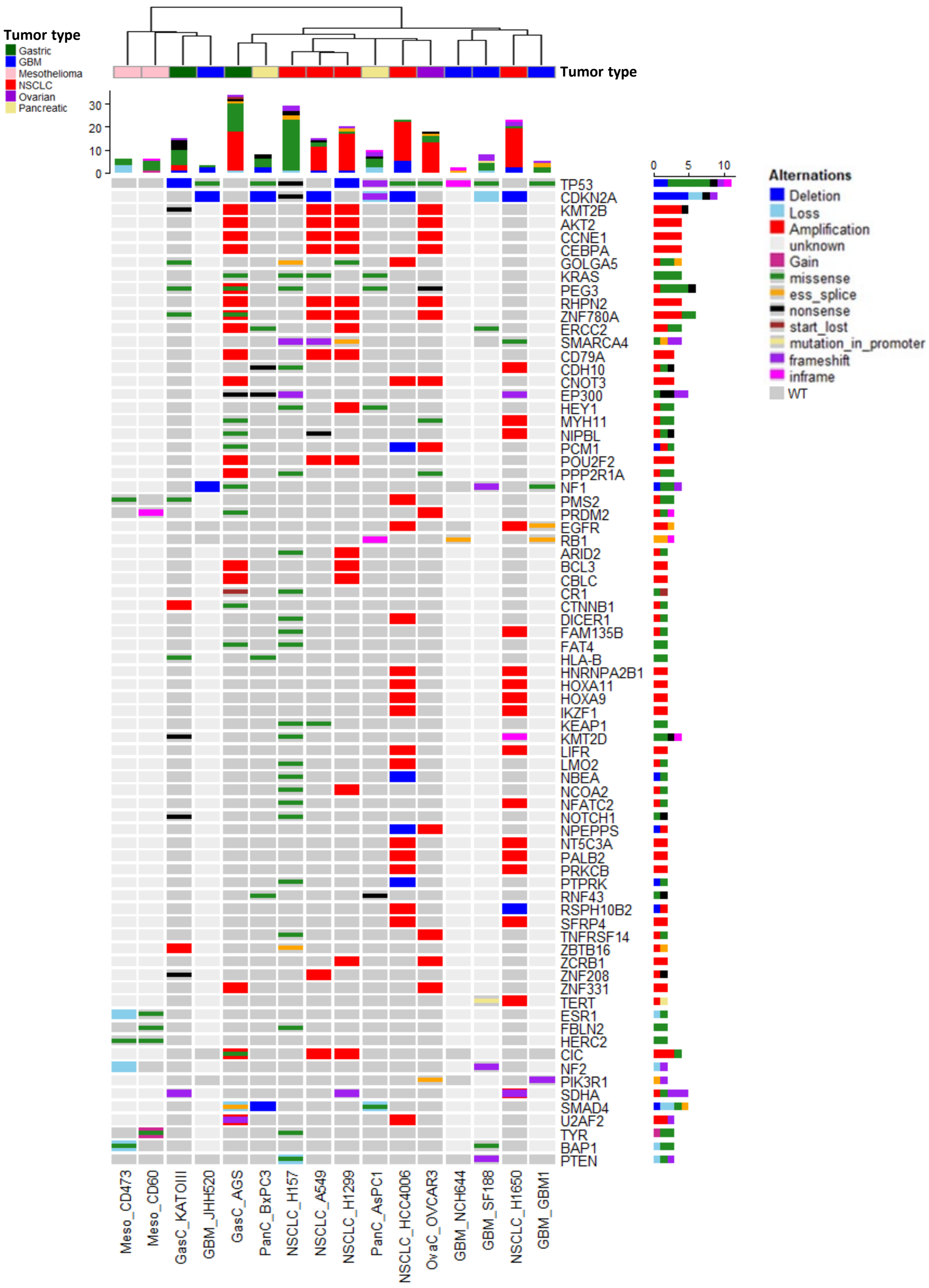

Figure S1. Cell line mutational status.

Oncoprint of cell lines mutation status. Cell lines are ordered according to **Fig. 3B**. Mutation types correspond to the right list. On the top there is the clustering according to **Fig. 2B**. The barplot on the top indicates the sum of mutations in these genes for these cell lines. The barplot on the right shows number of mutated datasets in each gene. Mutation definitions are as in https://cellmodelpassports.sanger.ac.uk/ .

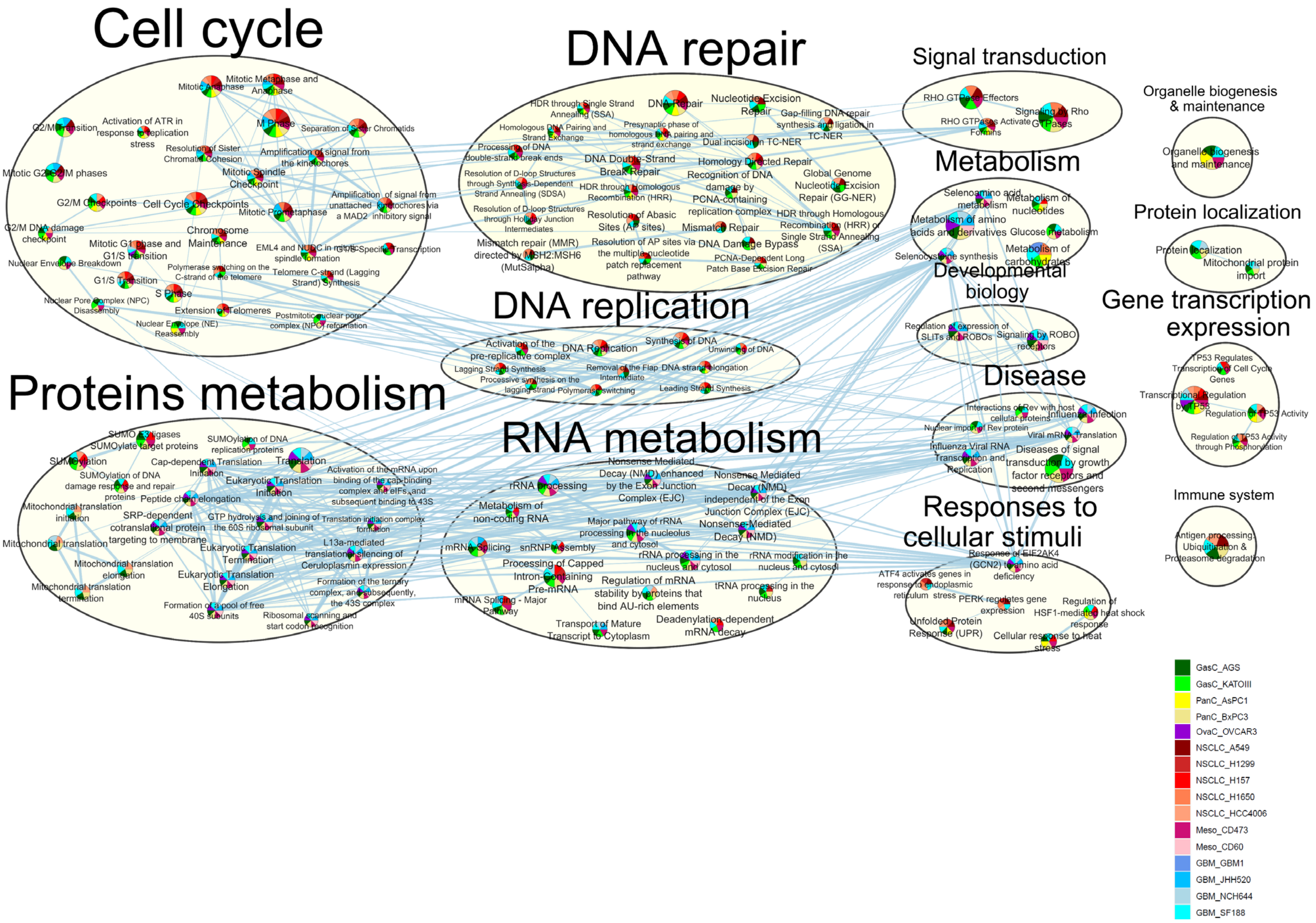

Figure S2. The complete enrichment map described in **Fig. 3A**.

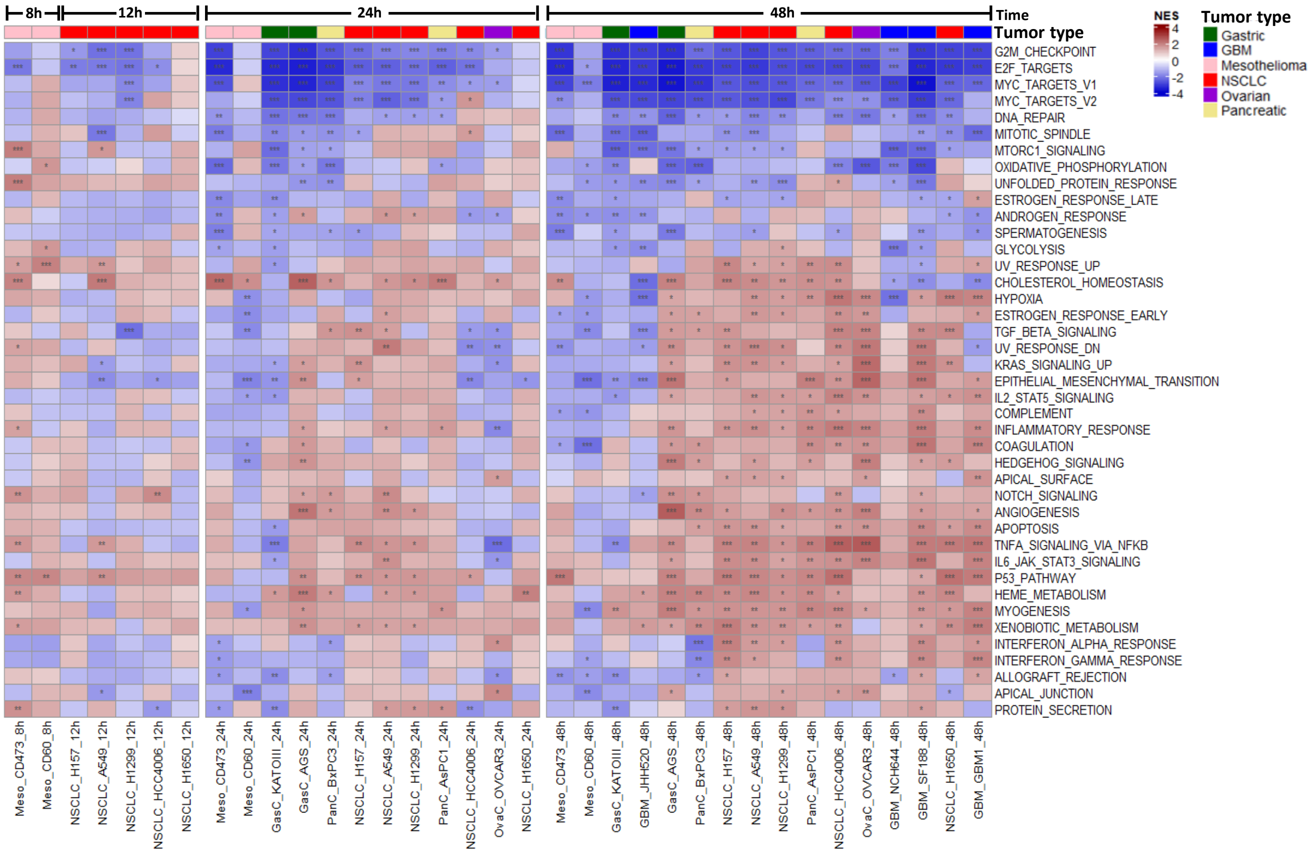

Figure S3. Supplementary data for **Figure 3B**.

GSEA heatmap performed with MsigDB hallmark gene sets for all datasets at all timepoints, showing pathways which were significant for at least 5 datasets at 48 h of treatment, arranged at each time point according to the hierarchical clustering of the 48-h treatment. Normalized Enrichment Score (NES): Red = upregulated, Blue = downregulated. False Discovery Rate (FDR): * FDR ≤ 0.05, ** FDR ≤ 0.01, *** FDR ≤ 0.001. Cell lines from each tumor type are labeled with a different color. The order of the datasets is according to the hierarchical clustering in **Fig. 3B**.

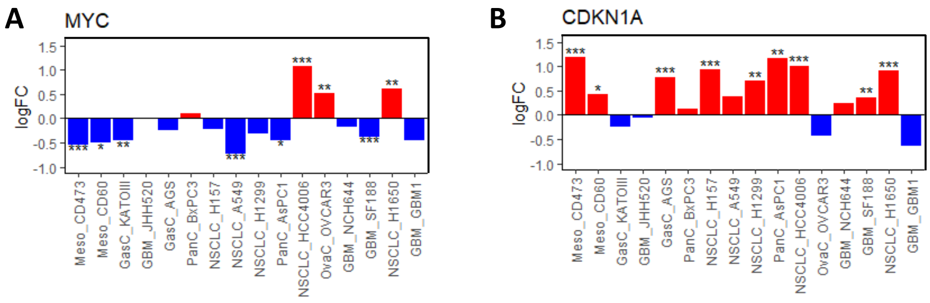

Figure S4. Log fold change for TTFields treatment versus control of MYC (A) and CDKN1A (B) expression across the various datasets. * p-value ≤ 0.05, ** p-value ≤ 0.01, *** p-value ≤ 0.001.
