# Supplementary material for: The transcriptomic fingerprint of cancer response to Tumor Treating Fields (TTFields)
Source: Cell Death Discov. 2025 Jul 10;11:319. doi: 10.1038/s41420-025-02615-5 (PMC12246047; doi:10.1038/s41420-025-02615-5)
Supplement: Supplementary file 4 — Table S3 [file 41420_2025_2615_MOESM4_ESM.docx]

**Table S3. Pathway categories and top functions of the IPA canonical pathways of Figure 3C**

| **Canonical Pathway** | **Pathway Categories** | **Top Functions** |
| --- | --- | --- |
|  |  |  |
| Cell Cycle Control of Chromosomal Replication | Cell Cycle Regulation | Connective Tissue Disorders; Developmental Disorder; Gastrointestinal Disease |
| EIF2 Signaling | Cellular Growth, Proliferation and Development; Cellular Stress and Injury; Intracellular and Second Messenger Signaling | Protein Synthesis; Gene Expression; Cell Death and Survival |
| NER (Nucleotide Excision Repair, Enhanced Pathway) | Cellular Stress and Injury | DNA Replication, Recombination, and Repair; Nucleic Acid Metabolism; Small Molecule Biochemistry |
| MicroRNA Biogenesis Signaling Pathway | Cellular Growth, Proliferation and Development | Gene Expression; Protein Synthesis; Infectious Diseases |
| Cyclins and Cell Cycle Regulation | Cell Cycle Regulation | Cell Cycle; Cancer; Cardiovascular Disease |
| Mismatch Repair in Eukaryotes | Cellular Stress and Injury | DNA Replication, Recombination, and Repair; Cancer; Gastrointestinal Disease |
| Estrogen-mediated S-phase Entry | Cell Cycle Regulation; Nuclear Receptor Signaling | Cell Cycle; Connective Tissue Development and Function; Embryonic Development |
| Kinetochore Metaphase Signaling Pathway | Cellular Growth, Proliferation and Development | Cell Cycle; Cellular Assembly and Organization; DNA Replication, Recombination, and Repair |
| Role of Tissue Factor in Cancer | Cancer | Cellular Movement; Cardiovascular System Development and Function; Organismal Development |
| Senescence Pathway | Cellular Growth, Proliferation and Development; Cellular Stress and Injury; Organismal Growth and Development | Cell Cycle; Cell Death and Survival; Organismal Injury and Abnormalities |
| Superpathway of Cholesterol Biosynthesis | Sterol Biosynthesis | Cardiovascular System Development and Function; Cell Morphology; Embryonic Development |
| BER (Base Excision Repair) Pathway | Cellular Stress and Injury | DNA Replication, Recombination, and Repair; Nucleic Acid Metabolism; Small Molecule Biochemistry |
| Mitotic Roles of Polo-Like Kinase | Cell Cycle Regulation | Cell Cycle; Cancer; Organismal Injury and Abnormalities |
| STAT3 Pathway | Cellular Growth, Proliferation and Development; Transcriptional Regulation | Cancer; Cellular Growth and Proliferation; Cellular Development |
| Cholesterol Biosynthesis II (via 24,25-dihydrolanosterol) | Sterol Biosynthesis | Free Radical Scavenging; Lipid Metabolism; Molecular Transport |
| Cholesterol Biosynthesis III (via Desmosterol) | Sterol Biosynthesis | Free Radical Scavenging; Lipid Metabolism; Molecular Transport |
| Cholesterol Biosynthesis I | Sterol Biosynthesis | Free Radical Scavenging; Lipid Metabolism; Molecular Transport |
| Autophagy | Cellular Stress and Injury; Organismal Growth and Development | Cell Morphology; Cellular Function and Maintenance; Cellular Assembly and Organization |
| Myelination Signaling Pathway | Cellular Growth, Proliferation and Development; Neurotransmitters and Other Nervous System Signaling | Nervous System Development and Function; Cellular Development; Tissue Development |
| CLEAR Signaling Pathway | Cellular Stress and Injury; Transcriptional Regulation | Cell Morphology; Cellular Function and Maintenance; Developmental Disorder |
| Aryl Hydrocarbon Receptor Signaling | Apoptosis; Cell Cycle Regulation; Ingenuity Toxicity List Pathways; Nuclear Receptor Signaling; Xenobiotic Metabolism | Gene Expression; Cell Cycle; Cell Death and Survival |
| ID1 Signaling Pathway | Cancer; Organismal Growth and Development; Transcriptional Regulation | Cancer; Organismal Injury and Abnormalities; Cellular Development |
| Role of BRCA1 in DNA Damage Response | Cancer; Cellular Stress and Injury | DNA Replication, Recombination, and Repair; Cancer; Hereditary Disorder |
| Cell Cycle: G1/S Checkpoint Regulation | Cell Cycle Regulation; Ingenuity Toxicity List Pathways | Cell Cycle; Cellular Growth and Proliferation; Connective Tissue Development and Function |
| Superpathway of Inositol Phosphate Compounds | Phospholipid Biosynthesis; Cyclitols Biosynthesis | Carbohydrate Metabolism; Lipid Metabolism; Small Molecule Biochemistry |
| HIF1α Signaling | Cardiovascular Signaling; Cellular Stress and Injury | Cell Cycle; Cell Death and Survival; Tissue Development |
| Adipogenesis pathway | Intracellular and Second Messenger Signaling | Cellular Development; Connective Tissue Development and Function; Tissue Development |
| Role of CHK Proteins in Cell Cycle Checkpoint Control | Cell Cycle Regulation; Cellular Stress and Injury | Cell Cycle; DNA Replication, Recombination, and Repair; Cellular Assembly and Organization |
| ATM Signaling | Cell Cycle Regulation; Cellular Stress and Injury | DNA Replication, Recombination, and Repair; Cell Cycle; Cellular Assembly and Organization |
