# Supplementary material for: The transcriptomic fingerprint of cancer response to Tumor Treating Fields (TTFields)
Source: Cell Death Discov. 2025 Jul 10;11:319. doi: 10.1038/s41420-025-02615-5 (PMC12246047; doi:10.1038/s41420-025-02615-5)
Supplement: Supplementary file 5 — Table S4 [file 41420_2025_2615_MOESM5_ESM.docx]

**Table S4. Role in cell and regulated proteins for the IPA upstream regulators of Figure 5A**

| **Upstream Regulator** | **Role in Cell** | **Regulates** |
| --- | --- | --- |
|  |  |  |
| TP53 | Apoptosis, Expression in, Proliferation, Cell cycle progression, Growth, Cell death, Senescence, G1 phase, Activation in, Transactivation in | CDKN1A, BAX, MDM2, BBC3, TP53, FAS, GADD45A, BCL2, CCNB1, PMAIP1, CCNG1, Pmaip1, BIRC5, reporter gene, APAF1 |
| MYC | Apoptosis, Proliferation, Transformation, Expression in, Growth, Cell cycle progression, Differentiation, Transcription in, S phase, Cell death | MYC, CDKN1A, TERT, CDKN1B, TP53, CCND1, CDK4, CDKN2A, NCL, FASLG, ODC1, CDC25A, MDM2, GADD45A, CAD |
| RABL6 | Expression in, Phosphorylation in, G1 phase, Cell death, Apoptosis, Growth, stabilization in, Ubiquitination in, G1/S phase Transition, Proliferation | CDK2, TP53, TOP2A, PRC1, CENPF, FRMD4A, PLK1, POLD1, MAD2L1, VRK1, RAD54B, H2AX, DRAM1, PRM2, PSME3 |
| CDKN2A | Apoptosis, Proliferation, Cell cycle progression, Senescence, Growth, Expression in, G1 phase, Binding in, Transformation, S phase | TP53, CDKN1A, MDM2, CDKN2A, RB1, CCND1, CDK4, E2F1, BAX, BCL2, VEGFA, CDK6, BIRC5, Cyclin A, TNF |
| CEBPB | Expression in, Differentiation, Transactivation in, Proliferation, Apoptosis, Activation in, Binding in, Transcription in, Adipogenesis, Morphology | IL6, PPARG, PTGS2, CEBPA, CEBPB, CXCL8, LCN2, DNA endogenous promoter, CRP, DNA promoter, PPARGC1A, CCNE1, HSD11B1, TNF, IL1B |
| Eldr | Transactivation in, Expression in, G2/M phase | FOXM1, CTCF, RPE65, BRCA1, GTSE1, ORC1, IQGAP3, ASPM, PLK4, KIF4A, NCAPG, ESPL1, DLGAP5, SPAG5, MYBL2 |
| CKAP2L | Growth, Expression in, Proliferation, Cell death | DYNC1I1, BUB1, RAB1A, NDC1, BIRC5, H2AZ2, ESPL1, CENPP, CKAP5, CENPA, TUBB4B, ZWINT, CDC20, SGO2, CENPE |
| TBX2 | Senescence in, Expression in, Proliferation, Repression in, Apoptosis, Differentiation, Immortalization, Lifespan, Fate determination, Phosphorylation in | DNA endogenous promoter, DNA promoter, RNA polymerase II, CDKN2A, CDKN1A, PTEN, CKAP2, TCEA2, NCAPD2, MCM4, HAUS1, SMC2, HEY1, ATF5, E2 |
| NUPR1 | Apoptosis, Expression in, Autophagy by, Cell cycle progression, Proliferation, Cell viability, Glycolysis in, Growth, Phosphorylation in, Processing in | FOXO3, TP53, BNIP3, DDIT3, CDK4, CDK2, TMEM158, NFIL3, ABL2, MMS22L, PER3, PHTF1, HFE, PARP9, ALG8 |
| E2f | G1/S phase transition, Cell cycle progression, Apoptosis, S phase, Proliferation, Differentiation, Cell division, Growth, Transformation, Transactivation in | CDC6, synthetic promoter, CCNE1, Cyclin E, reporter gene, STMN1, RRM2, DHFR, E2F1, Cyclin A, CDC7, Cdk (family), MCM2, MYC, CDK2 |
| EP400 | Loss, Expression in, Apoptosis, Cell cycle progression, G2/M phase, Generation, Polyploidization, S phase, Growth, Lack | TP53, CDKN1A, MYBL2, RASSF1, CCNA2, FOXM1, CDC25A, E2F7, CCNF, SKP2, E2F8, RBL1, CDCA3, PLK1, SGO1 |
| KDM1A | Expression in, Differentiation, Activation in, Methylation in, Binding in, Dimethylation in, Accumulation in, Transactivation in, Ubiquitination in, Destabilization in | CDH1, SOX2, CDKN1A, VIM, CXCL10, LCP1, PTEN, ELOVL7, TM4SF1, CD274, GFAP, DNA promoter, Gamma globin, SREBF1, FASN |
| TNFRSF9 | Proliferation, Expansion, Expression in, quantity, Survival, Deletion, Activation in, Activation, Degradation in, Function | IFNG, TNF, IL4, IL2, BCL2L1, Pro-inflammatory Cytokine, cytokine, IL13, IL12 (family), NFkB (complex), IgG2a, CD80, IL6, CD14, PCNA |
| FOXM1 | Expression in, Proliferation, Growth, Activation in, Phosphorylation in, Mitosis, Transactivation in, Formation in, Transcription in, Senescence | CCNB1, CDC25B, CDKN1A, MMP2, AURKB, PLK1, SKP2, BIRC5, CENPA, CCNA2, CCND1, CDKN1B, CCNB2, CDKN2A, CDC20 |
| CSF2 | Differentiation, Proliferation, Apoptosis, Expression in, Activation, Survival, Quantity, Stimulation, Generation, Growth | CD86, CD14, CD80, CD40, TNF, IL1B, STAT5a/b, BCL2L1, MHC Class II (complex), ICAM1, CD1A, IL6, BIRC5, IL10, BCL2 |
| TGFB1 | Expression in, Phosphorylation in, Proliferation, Epithelial-mesenchymal transition, Binding in, Migration, Differentiation, Activation in, Apoptosis, Growth | ACTA2, SERPINE1, FN1, SMAD3, CDH1, COL1A1, CDKN1A, SMAD2, CCN2, VIM, SMAD7, SNAI1, FOXP3, CDKN2B, Collagen Alpha1 |
| CD3E | Proliferation, Apoptosis, Activation, Differentiation, Lack, Development, Abnormal morphology, Quantity, Expression in, Co-stimulation | IL2, IFNG, IL4, ZAP70, FASLG, TNF, CD69, IL2RA, MAPK3, MAPK1, PLCG1, ERK1/2, CD247, CD3E, IL10 |
| TOX | Number, Differentiation, Expression in, Commitment, Abnormal morphology, Development, Colony formation by, Cell cycle progression, Activation in, Cell viability | ID2, Tcf7, EZH2, KLF2, CDKN2C, KMT2B, TFDP1, RPA2, EOMES, EP300, KMT2C, RBL1, BUB3, CREBBP, C |
| MYCN | Expression in, Proliferation, Apoptosis, Transactivation in, Transcription in, Transformation, Cell death, Recruitment in, Activation in, Growth | MYCN, ITGB1, ABCC1, CAV1, TP53, HMGA1, NGFR, MXI1, Focal adhesion kinase, ITGA3, ITGA2, HDAC2, NME1, RPL10, CCND1 |
| RBL2 | Proliferation, G1 phase, Expression in, Senescence, Growth, Cell cycle progression, Differentiation, Apoptosis, Binding in, Anoikis | E2F1, PLK1, RBL2, MYBL2, CCNE1, Cyclin A, BUB1, CCNF, CCNB1, RB1, CDKN2A, CCND1, Cyclin E, MYOD1, Cyclin a |
| LARP1 | Translation in, Proliferation, Synthesis in, Binding in, Macroautophagy in, Fitness, Stabilization in, Invasion, Growth, Replication in | RPL32, RPS20, RPS6, PABPC1, PABP, RPS18, RPS11, RPS19, RPL23A, RPL11, RPS3A, RPS15A, RPS5, RPL7A, RPS27A |
| EGF | Phosphorylation in, Proliferation, Activation in, Migration, Expression in, Binding in, Growth, Apoptosis, Stimulation, Signaling in | EGFR, ERK1/2, Akt, MAPK1, Mapk, ERK, STAT3, MAPK3, FOS, SHC1, PI3K (complex), RAF1, ERBB2, JUN, SRC |
| TFEB | Expression in, Response, Organization, Biogenesis, Number, Autophagy by, Response by, Function, Quantity, Apoptosis | CTSD, CTSB, TFEB, SQSTM1, MCOLN1, CTSF, CCND1, MYC, CDH1, LAMP1, IL1B, Vacuolar H+ ATPase, ATG5, SGSH, VPS35 |
